# Mathematical Modeling of Atmospheric Effects on Distance Determination Accuracy in the VDES R-Mode System

**DOI:** 10.3390/s26103127

**Published:** 2026-05-15

**Authors:** Krzysztof Bronk, Patryk Koncicki, Adam Lipka, Rafal Niski, Blazej Wereszko

**Affiliations:** National Institute of Telecommunications, 04-894 Warsaw, Poland; k.bronk@il-pib.pl (K.B.); a.lipka@il-pib.pl (A.L.); r.niski@il-pib.pl (R.N.); b.wereszko@il-pib.pl (B.W.)

**Keywords:** GNSS alternatives, maritime radiocommunications, ranging, e-navigation, R-Mode Baltic, VHF data exchange system, mathematical modeling

## Abstract

This paper investigates the impact of atmospheric conditions on distance determination accuracy in the VDES R-Mode system, based on system development and long-term analytical work conducted within the ORMOBASS project. A dedicated VDES R-Mode transmitter and monitoring station were developed and deployed in Poland, in the Port of Gdynia and at the boatswain’s office in the port of Jastarnia, respectively. Both stations were synchronized in time and frequency using a fiber-optic link and White Rabbit technology, ensuring high-precision and stable operation during long-term measurements. Based on a one-year stationary measurement campaign, a comprehensive dataset combining ranging results and meteorological observations was collected and analyzed. Statistical evaluation demonstrated that atmospheric conditions—particularly rainfall intensity and water vapor density—have a measurable impact on ranging accuracy. These findings motivated the development of a mathematical model describing the relationship between atmospheric conditions and distance measurement errors. The proposed logarithmic regression-based approach was validated using real measurement data; the authors also demonstrated its ability to reduce error variability during changing weather conditions, indicating its potential for future implementation in VDES R-Mode receivers.

## 1. Introduction

Navigation has become an indispensable component of modern life, underpinning a vast array of critical applications across sectors such as transportation, logistics, agriculture, telecommunications, and emergency response. Nowhere is this more evident than in maritime operations, where precise positioning, navigation, and timing (PNT) information is vital for safety, efficiency, and coordination at sea. Global Navigation Satellite Systems (GNSSs), particularly the Global Positioning System (GPS), have long served as the backbone of modern navigation infrastructure. However, the increasing reliance on GNSSs has revealed a significant vulnerability: susceptibility to both intentional and unintentional interference. Due to the very low signal levels reaching Earth, the GNSS signals are exceptionally susceptible to interference, including intentional jamming, spoofing, and meaconing [1].

In recent years, concerns over GNSS resilience have intensified, especially in the wake of Russia’s full-scale invasion of Ukraine in 2022. This conflict has underscored the strategic importance of PNT systems and the ease with which they can be disrupted. Instances of GNSS jamming and spoofing have surged not only in conflict zones but also across broader regions, including the Baltic Sea area. There, ships, aircraft, and other GNSS-reliant platforms have frequently reported signal loss or manipulation, with a growing body of evidence pointing to Russian-originated interference. These disruptions not only compromise safety and operational effectiveness but also expose the limitations of relying solely on satellite-based navigation systems.

Given the critical role of reliable navigation in maritime operations, the urgent need for a robust and independent alternative to GNSSs has become increasingly clear, particularly in the context of resilient PNT strategies and secure terrestrial backup solutions currently emphasized at the European operational level [2]. One of the most promising solutions in this context is the R-Mode (Ranging Mode) system, which provides a strategic and technological response to these evolving threats. R-Mode is intended to operate as a ground-based backup system for satellite-based PNT solutions such as GPS, Galileo, or GLONASS. Similar to GNSSs, the R-Mode system provides PNT information that can support vessel navigation and maneuvering, particularly in coastal waters and port areas. However, unlike GNSSs, R-Mode is entirely independent of the satellite segment. Instead, it relies on a terrestrial network of reference radio stations and determines position using the Time of Arrival (TOA) method applied to known transmitted signals.

In general, the R-Mode concept can be implemented using two types of reference radio stations:

- Stations operating in the MF band (e.g., DGPS stations), referred to as the MF R-Mode component [3];
- Stations operating in the maritime VHF band, including VDES [4] or the existing AIS infrastructure, referred to as the VDES/AIS R-Mode component.

In this article, the focus is placed on the VDES R-Mode component. The VDES system is a digital telecommunications system for maritime applications utilizing VHF base stations located close to the shore. VDES R-Mode is an opportunistic PNT solution based on the terrestrial part of the VDES system that uses the existing marine data transmission system for navigation purposes. The authors have extensive experience in the field of VDES and R-Mode system development and evaluation. Their early work included development and analysis of a dedicated physical-layer simulator for the terrestrial VDES component, published in [5], as part of the EfficienSea2 project [6]. In subsequent years, the authors conducted and reported comprehensive simulation and measurement studies, including real-world AIS and VDES measurement campaigns, as well as the conceptual and experimental development of the R-Mode Baltic system [7,8,9].

From a broader perspective, environmental effects on radio-based positioning and communication systems are well understood in other domains. In satellite navigation systems such as GPS and Galileo, atmospheric propagation effects are routinely mitigated using established correction models, including tropospheric delay models [10,11] and ionospheric correction approaches such as the widely used Klobuchar model [12]. Similarly, for terrestrial communication systems, numerous empirical channel and propagation models have been developed for technologies such as Wi-Fi, accounting for environmental attenuation, shadowing, and multipath effects [13].

In contrast, systematic modeling of atmospheric influences on terrestrial radionavigation systems operating in the maritime VHF band remains limited. In this context, the present work extends the authors’ earlier experience in VHF channel modeling and VDES system analysis by proposing a regression-based model tailored to maritime radionavigation and ranging applications, thereby addressing a gap between satellite-domain correction models and terrestrial communication channel models.

While the feasibility of VDES R-Mode positioning has been demonstrated in multiple studies and pilot deployments [14,15], the achievable ranging accuracy remains sensitive to radio-wave propagation conditions. Atmospheric factors such as precipitation and tropospheric moisture can introduce systematic variations in signal delay, leading to increased distance determination errors. Already during short-term stationary measurement campaigns conducted within the R-Mode Baltic-2 project [16], the authors observed a clear impact of atmospheric precipitation on the distance determination accuracy of the VDES R-Mode system, as reported in [17]. A common approach to mitigating such effects is continuous system monitoring using dedicated reference receivers, which enables real-time error estimation and correction. However, the deployment and maintenance of permanent monitoring infrastructure significantly increases system complexity and operational costs.

This raises a fundamental question: whether it is possible to mitigate a substantial part of atmospheric-induced ranging errors using mathematical models derived from long-term observations, rather than relying exclusively on continuous real-time monitoring. If such models could reliably represent the relationship between atmospheric conditions and ranging accuracy, they could be implemented directly within R-Mode receivers as correction mechanisms, reducing infrastructure requirements and lowering overall system costs.

Motivated by this perspective, the present work investigates whether a mathematical model can be developed to describe and predict the influence of atmospheric conditions on distance determination accuracy in the VDES R-Mode system. To address this problem, long-term stationary measurements were conducted and analyzed, providing a comprehensive dataset that captures a wide range of environmental conditions. Based on these measurements, statistical analyses and regression-based modeling were performed to assess the feasibility of representing atmospheric effects through a compact mathematical formulation.

This paper comprises five main section (excluding this Introduction). In Section 2, a general overview of the VDES R-Mode testbed is provided, including its architecture and functionality. It was implemented by the authors and comprises an R-Mode base station and an R-Mode monitoring station. The testbed was responsible for collecting the data the eventual model was based on. Section 3 introduces the assumptions and organization of the data collection process which spanned exactly one full year, from October 2024 to October 2025. Section 4 is dedicated to a thorough analysis of the collected data, particularly the ranging accuracy errors observed for various meteorological conditions. The results of this analysis served as a foundation for the logarithmic regression-based mathematic model describing the relationship between atmospheric conditions and distance measurement errors in the VDES R-Mode system. The model itself is presented in details and discussed in Section 5. Finally, all the achievements and main aspects discussed in the article are revisited in Section 6—in the form of conclusions.

## 2. VDES R-Mode Testbed in Poland

The measurement infrastructure used in this study was developed within successive Baltic Sea R-Mode implementation projects, culminating in the ongoing ORMOBASS operational monitoring campaign [16,18,19]. These initiatives enabled the construction of a functional VDES/MF R-Mode testbed used for long-term ranging analyses.

The Polish part of the R-Mode testbed was developed by the National Institute of Telecommunications and currently consists of two main elements:

- An R-Mode base station demonstrator installed on an antenna tower in the Port of Gdynia;
- An R-Mode monitoring station located on the roof of the boatswain’s office building in the port of Jastarnia.

These VDES R-Mode stations were initially constructed and expanded within the R-Mode Baltic and R-Mode Baltic-2 projects and are currently being further developed and tested as part of the ORMOBASS project.

### 2.1. VDES R-Mode Base Station in Gdynia

The VDES R-Mode base station was installed on 19 September 2024 in the Port of Gdynia, within the facilities of the Maritime Office in Gdynia (MOG). The station equipment is located in a building supervised by the MOG, while the transmitting antenna is mounted on a nearby tower at a height of approximately 28 m above mean sea level. The effective isotropic radiated power (EIRP) of the transmitting antenna is 25 W, which is a typical value for maritime VHF shore-based transmitters. Following the installation, the VDES R-Mode transmitter was commissioned on site, and initial operational tests were performed by the National Institute of Telecommunications.

**Figure 1:**
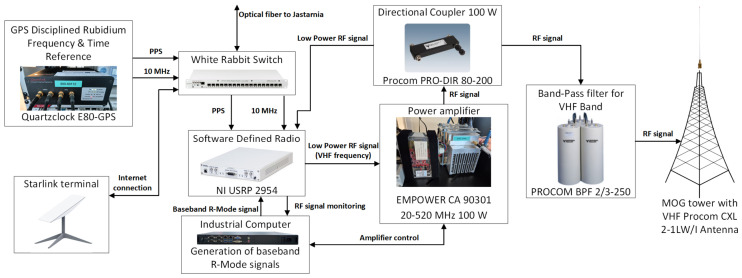
VDES R-Mode base station block diagram (Gdynia Port).

Figure 1 presents a block diagram of the VDES R-Mode base station installed in Gdynia.

All hardware components of the constructed VDES R-Mode transmitting station are listed below, together with a brief description of their primary functions:

- Starlink terminal (SpaceX, Hawthorne, CA, USA)—provides an Internet connection and enables remote management and monitoring of station operation, as well as data transfer to NIT for analysis and archiving of measurement results;
- GPS-disciplined rubidium frequency and time reference, Quartzclock E80-GPS (Quartzlock Ltd., London, UK)—provides stable reference frequency and time for synchronous triggering of signal transmission and reception; the rubidium oscillator operates in free-running mode without GPS disciplining to improve long-term stability (as studies performed by the authors in the R-Mode and R-Mode 2 projects have shown that disciplining the oscillator is a source of additional error [9,17]);
- White Rabbit low-jitter switch, WRS-3-LJ/18 (Seven Solutions, Granada, Spain)—used for time and frequency transfer between geographically separated devices (stations in Gdynia and Jastarnia); further details are provided in Section 2.4;
- Software-defined radio (SDR)—NI USRP 2954 (National Instruments, Austin, TX, USA)—generates a low-power RF VDES R-Mode signal from the baseband signal provided by the industrial computer;
- Industrial computer—Sigma S1U (OnLogic, South Burlington, VT, USA)—generates baseband VDES R-Mode signals and controls and supervises the operation of all system components;
- Directional coupler (100 W)—Procom PRO-DIR 80–200 (Procom A/S, Frederikssund, Denmark)—enables monitoring of the transmitted RF signal;
- Power amplifier (Empower RF Systems, Inglewood, CA, USA)—EMPOWER CA 90301 (20–520 MHz, 100 W)—amplifies the RF signal to the required output power;
- VHF band-pass filter—PROCOM BPF 2/3–250 (Procom A/S, Frederikssund, Denmark)—limits out-of-band emissions generated on the transmitting side;
- VHF antenna—Procom CXL 2-1LW/I (Procom A/S, Frederikssund, Denmark)—omnidirectional antenna installed on top of the tower in the Port of Gdynia.

### 2.2. VDES R-Mode Monitoring Station in Jastarnia

**Figure 2:**
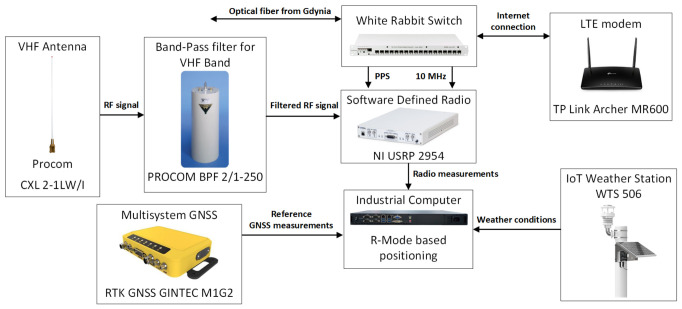
VDES R-Mode monitoring station block diagram (Jastarnia boatswain’s office).

The VDES R-Mode monitoring station was installed on 26 September 2024 at the boatswain’s office in the port of Jastarnia, approximately 19.9 km from the transmitting station. The theoretical maximum distance for maintaining line-of-sight (LOS) conditions can be estimated using the well-known radio horizon formula from [9]:

(1) $$d_{LOS}=4.12\cdot (\sqrt{h_{tx}}+\sqrt{h_{rx}})$$

where $h_{tx/rz}$ is the antenna height of the transmitter and receiver, respectively. If we substitute $h_{tx}$ = 28 m a.m.s.l. and $h_{rx}$ = 17 m a.m.s.l. into Equation (1), we can calculate the theoretical radio horizon limit as $d_{LOS}$ = 38.8 km. The radio propagation path is entirely over the sea and under line-of-sight (LOS) conditions. The station is located within the facilities of the Maritime Office in Gdynia; the receiver equipment is installed inside a building supervised by the MOG, while the VHF antenna is mounted on the roof of the boatswain’s office building in the port of Jastarnia at a height of approximately 17 m above mean sea level. Following the installation, the VDES R-Mode receiver was commissioned on site, and initial system operation tests were conducted by the National Institute of Telecommunications. Figure 2 presents a block diagram of the VDES R-Mode monitoring station installed in Jastarnia.

All hardware components of the constructed VDES R-Mode receiving station are listed below, together with a brief description of their primary functions:

- VHF antenna—Procom CXL 2-1LW/I—omnidirectional antenna installed on the roof of the boatswain’s office building in the port of Jastarnia.
- VHF band-pass filter—PROCOM BPF 2/1-250 (Procom A/S, Frederikssund, Denmark)—limits out-of-band interference on the receiving side.
- White Rabbit low-jitter switch (WRS-3-LJ/18)—used for time and frequency transfer between geographically separated devices (stations in Gdynia and Jastarnia); further details are provided in Section 2.4.
- Software-defined radio (SDR)—NI USRP 2954—converts the received low-power RF VDES R-Mode signal into a baseband signal and forwards it to the industrial computer.
- Industrial computer—Sigma S1U—collects baseband VDES R-Mode signals and controls and supervises the operation of all system components.
- Multisystem GNSS receiver—RTK GNSS GINTEC M1G2 (Gintec, Taipei, Taiwan)—provides a reference position for system verification purposes.
- IoT weather station—WTS 506 (Milesight IoT Co., Ltd., Xiamen, China)—records meteorological parameters relevant to propagation analysis.
- LTE modem—TP-Link Archer MR600 (TP-Link Technologies Co., Ltd., Shenzhen, China)—provides an Internet connection and enables remote management and monitoring of station operation, as well as data transfer to NIT for analysis and archiving of measurement results.

As part of the long-term stationary measurements, all signal samples were recorded and stored in binary format using the USRP module (NI USRP 2954). The measurement software, implemented in the NI LabVIEW 2018 SP1 environment, was adapted for continuous autonomous operation between the transmitting station in Gdynia and the receiving station in Jastarnia.

### 2.3. Radio Link Budget Calculations

The expected ranging accuracy for the current Gdynia–Jastarnia radio link can be calculated, as shown in [9], as a function of the signal-to-noise ratio (SNR) and the effective signal bandwidth using the Cramer–Rao lower bound method. Using the radio link budget calculation, it is possible to predict the expected SNR value, important for further calculations.

The expected received signal level can be calculated on the basis of Equation (2), assuming the transmitter power level and propagation loss on the Gdynia–Jastarnia radio link. The propagation loss calculation can be conducted using the well-known ITU-R P.1546-6 propagation model [20]. On the basis of the analysis of propagation conditions for the marine VHF communications systems operating in the Baltic Sea area, presented in [21], the sea path model calculation for the investigated radio link should be corrected by the 16 dB factor due to low salinity of the Gulf of Gdansk.

(2) $$P_{RX}=P_{TX}+G_{TX}-L_{TX}-L_{1546}-L_{cor}+G_{RX}-L_{RX}$$

where $P_{RX}$ is the expected received signal level, $G_{TX}$ is the transmitter antenna gain (2.15 dBi), $L_{TX}$ is the transmitter losses (sum of the VHF filter attenuation 3 dB and RF cable attenuation 5 dB), $L_{1546}$ is the ITUR P.1546-6 propagation loss (119.8 dB), $L_{cor}$ is the sea path model correcting factor (16 dB), $G_{RX}$ is the receiver antenna gain (2.15 dBi), and $L_{RX}$ is the receiver losses (sum of the VHF filter attenuation 1 dB and RF cable attenuation 2 dB). Based on above, the expected received signal level $P_{RX}$ equals −92.65 dBm.

The expected noise power level *P_N_* can be calculated on the basis of Equation (3) as the thermal noise level for the 100 kHz bandwidth accumulated with the human-made noise, obtained from the ITU-R P.372-17 recommendation [22].

(3) $$P_{N}=N_{0}+10\cdot {log}_{10}\left(B\right)+F_{mm}$$

where $N_{0}$ is noise power spectral density (−174 dBm/Hz at 290 K), *B* is the signal bandwidth (100 kHz), and $F_{mm}$ is the human-made noise factor (16 dB). Based on above, the expected noise level *N* equals −108 dBm.

The expected signal-to-noise ratio can be calculated on the basis of the following equation:

(4) $$SNR=P_{RX}-P_{N}$$

Based on above, the expected signal-to-noise ratio equals 15.35 dB. This means that the theoretical ranging accuracy for this SNR value should be approximately 5.74 m according to the Cramér–Rao lower bound (CRLB) method [9]. For the conducted measurement campaign, however, the recorded median SNR was 12.9 dB, which theoretically corresponds to an accuracy of approximately 7.61 m. In practice, for this SNR level, under non-precipitation atmospheric conditions and low humidity, an accuracy on the order of approximately 8 m was obtained.

### 2.4. Synchronization of VDES R-Mode Stations in Gdynia and Jastarnia

One of the objectives of the ORMOBASS project is to evaluate time and frequency synchronization of VDES R-Mode stations using a fiber-optic link. This is one of the conclusions of the work within the previous R-Mode and R-Mode 2 projects, in which rubidium oscillators with GPS disciplining were used for synchronization. Measurement studies have shown that synchronizing the clock to UTC time was itself a source of additional error [9,17]. In order to eliminate that, an optical fiber connection between Gdynia and Jastarnia was established in cooperation with the Maritime Office in Gdynia (MOG), using the existing infrastructure. The fiber-optic network from Gdynia to Hel is routed across the Gulf of Gdańsk on the seabed and continues from Hel along the coastal road to Jastarnia and Rozewie.

**Figure 3:**
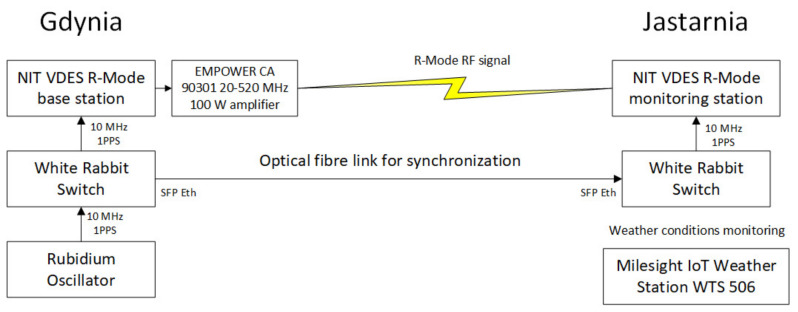
General architecture of the VDES R-Mode demonstrator with White Rabbit-based synchronization between the Gdynia base station and the Jastarnia monitoring station.

Figure 3 presents the general architecture of the VDES R-Mode demonstrator, in which synchronization between stations is achieved using the White Rabbit solution installed at both ends of the fiber-optic link and integrated with the Maritime Office in Gdynia infrastructure.

A synchronization experiment was conducted between the VDES R-Mode stations in Gdynia and Jastarnia. Time and frequency transfer was implemented using a fiber-optic connection, with the White Rabbit solution selected as the synchronization technique. The Maritime Office in Gdynia (MOG) provided access to the necessary infrastructure, enabling synchronization tests over a dedicated dark fiber link of approximately 40 km between the two locations and measurement of the established fiber optic connection.

Taking into account the requirements defined within the ORMOBASS project, as well as economic and technical constraints, atomic time references based on the rubidium standard were selected for use in the shore-based stations. This choice was motivated by their favorable long-term stability and low phase-noise characteristics. Although cesium and hydrogen standards offer superior long-term frequency stability, their high cost, large physical dimensions, significant power consumption, and strict environmental requirements make their practical deployment challenging.

Moreover, in time-transfer applications without the need for a global time reference such as UTC, short-term stability and low jitter are of primary importance. Under these conditions, rubidium frequency standards provide better overall performance than cesium oscillators. Consequently, the originally planned cesium-based solution was replaced with a rubidium-based time reference.

To achieve the lowest possible jitter, the following measures were adopted:

1. A rubidium oscillator was selected instead of a cesium oscillator.
2. The Quartzclock E80-GPS rubidium oscillator was equipped with a low phase-noise option, resulting in reduced jitter.
3. White Rabbit switches with a low-jitter configuration were selected.
4. The Quartzclock E80-GPS rubidium oscillator was operated in free-running mode, which further reduces jitter.

The experiment was designed to demonstrate the capability of providing network time with the accuracy and precision required by the R-Mode system. It also enables a performance comparison with solutions based on GPS-disciplined rubidium clocks deployed at both stations.

Most importantly, the experiment enables an assessment of the VDES R-Mode radio link performance under conditions in which the dominant non-propagation error sources affecting positioning accuracy were either eliminated or significantly reduced. As a result, the observed ranging errors can be primarily attributed to atmospheric and meteorological effects. Several key error sources that were intentionally minimized are shown below:

- Elimination of clock-related errors—fiber-optic synchronization between the transmitting and receiving stations allowed complete removal of time-drift effects and long-term clock instability. Consequently, the measurements were not affected by oscillator offsets or synchronization bias.
- Controlled and deterministic system geometry—the use of a single transmitting and a single receiving station ensured a fixed and well-defined geometry. The influence of geometric dilution of precision (GDOP) is well known and extensively described in the literature [23]; therefore, geometry was not treated as a variable in this study.
- Receiver noise characterization and control—the receiver noise figure was experimentally verified and incorporated into the link budget calculations. The theoretical relationship between signal-to-noise ratio and ranging accuracy is well established [9], allowing the noise contribution to be quantified and separated from propagation-related effects.
- Reduction in large-scale fading—the experiment was conducted over a predominantly maritime path with stable propagation characteristics, limiting terrain-induced shadowing and large-scale attenuation variability.
- Maintained line-of-sight (LOS) conditions—the transmitter and receiver heights ensured geometric visibility over the 19.9 km path, significantly reducing diffraction losses and improving signal stability.
- Static measurement configuration—both stations were fixed, eliminating Doppler effects, small-scale fading, and dynamic geometry variations that typically affect maritime positioning systems.

Due to the above measures, the remaining variability in the measured positioning accuracy can be attributed predominantly to atmospheric propagation phenomena. This controlled configuration therefore enables reliable investigation of the influence of meteorological parameters on VDES R-Mode positioning accuracy.

As a first step, stability tests of the rubidium oscillator (Quartzclock E80-GPS) operating in free-running mode (without GPS disciplining) were conducted. The stability of the generated frequency and time reference signal was measured using a high-performance digital oscilloscope (MXR604A Infinium MXR-Series, Keysight Technologies, Santa Rosa, CA, USA). The results showed a jitter level of approximately 225 ps, which represents a very good performance and serves as a reference level prior to the deployment of the White Rabbit solution.

The White Rabbit is a deterministic Ethernet-based network technology designed for high-precision time and frequency distribution. The White Rabbit switch (WRS) provides sub-nanosecond accuracy and picosecond-level precision for systems distributed over large distances. The WRS is a core component of the White Rabbit network for ultra-precise time and event distribution and was originally developed within the White Rabbit project at CERN [24].

**Figure 4:**
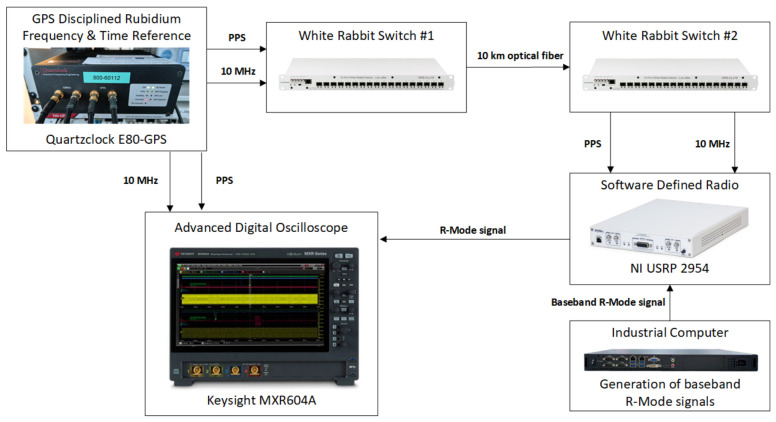
Diagram of the jitter measurement of the reference time signal from the Rubidium oscillator transferred via White Rabbit and the corresponding R-Mode RF output signal.

Under laboratory conditions, the stability of the R-Mode signal in RF band between two stations synchronized using 10 km optical fiber, 1550 nm SFP Transceivers, and the White Rabbit system was evaluated. The measurement stand was set up as follows: the PPS (Pulse Per Second) signal from the rubidium oscillator operating in free mode was fed to the first White Rabbit switch, and then, using a 10 km fiber optic cable and 1550 nm SFP transceivers, the PPS signal was transmitted to the second White Rabbit switch. The PPS signal transmitted via optical fiber was then supplied to the USRP module to trigger the transmission of the R-Mode signal. Figure 4 shows a diagram of the laboratory setup for measuring the jitter of a reference time signal. Measurements were conducted between the time reference signal (PPS from rubidium oscillator) transferred via the White Rabbit link and the corresponding R-Mode RF output signal. A stability level of approximately 480 ps was achieved after 20 h of continuous measurements, which corresponds to a distance determination accuracy of about 15 cm.

The base station installed in the Port of Gdynia uses a rubidium oscillator for synchronization. This same oscillator provides the reference signal for the station in Jastarnia. In this case, this signal is transmitted via the Gdynia Maritime Office’s fiber-optic cable (connecting Gdynia and Jastarnia) using the White Rabbit system.

## 3. Long-Term Stationary Measurements

The long-term stationary measurements were conducted to continuously monitor the performance and stability of the VDES R-Mode system over an extended period under real environmental conditions. The primary objective of these observations was to evaluate the influence of varying meteorological and propagation factors—such as temperature, humidity, air pressure, and rainfall—on the accuracy of distance determination between the transmitting station in Gdynia and the monitoring station in Jastarnia.

This stage of the experiment provided a valuable dataset for statistical and correlation analyses, forming the basis for developing a mathematical model describing the impact of atmospheric conditions on ranging accuracy. The long-term character of the measurements allowed for identifying seasonal and diurnal variations, verifying synchronization stability between stations, and validating the robustness of the VDES R-Mode system in continuous operation.

### 3.1. Measurement Campaign Scenario

The measurement campaign conducted within the ORMOBASS project aimed to evaluate the performance of the VDES R-Mode system under real environmental conditions. The measurements were carried out continuously from October 2024 to October 2025.

The test configuration consisted of two fixed sites: a transmitting station located in the Port of Gdynia and a monitoring station located in Jastarnia. The distance between the sites was approximately 19.9 km, with a propagation path entirely over the sea and under line-of-sight conditions. The transmitting antenna in Gdynia was installed at a height of approximately 28 m a.m.s.l., while the receiving antenna in Jastarnia was positioned at approximately 17 m a.m.s.l.

**Figure 5:**
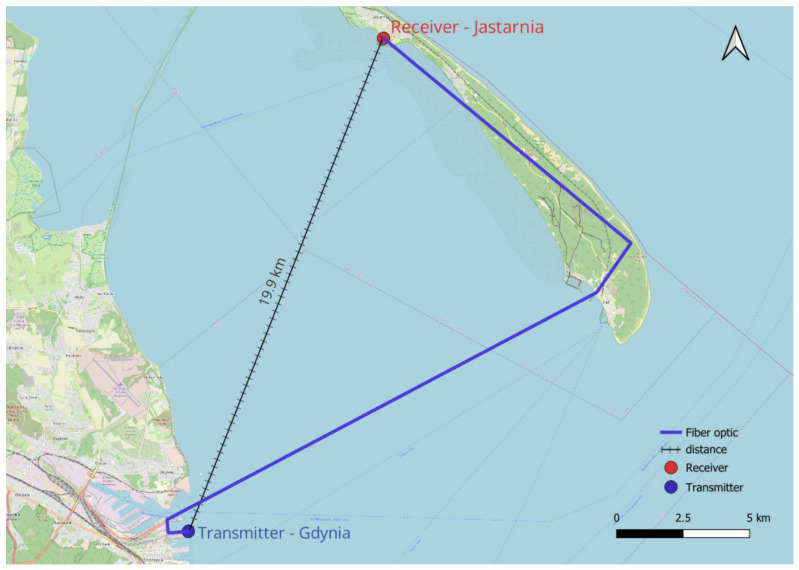
Measurement campaign scenario showing the location of the VDES R-Mode transmitting station in Gdynia and the monitoring station in Jastarnia.

The geographical arrangement of the transmitting and monitoring stations, together with radio propagation path, is illustrated in Figure 5, which presents the measurement campaign scenario adopted in this study.

Both stations were synchronized in time and frequency using the White Rabbit system, ensuring nanosecond-level precision. This synchronization allowed the analysis of signal arrival times and distance measurement errors. The monitoring station in Jastarnia was equipped with a Milesight WTS506 weather station (Milesight IoT Co., Ltd., Xiamen, China) [25] used for recording meteorological parameters relevant to propagation analysis, including air temperature, humidity, atmospheric pressure, wind speed, wind direction, and rainfall. The adopted configuration enabled long-term observation of the system’s stability and the collection of data required for further statistical analysis and model development.

### 3.2. Characteristics and Statistical Analysis of the Collected Measurement Data

As part of the ORMOBASS project, an extensive dataset was collected, covering both the parameters of the VDES R-Mode signal and meteorological observations recorded at the monitoring station in Jastarnia. The observation period spanned a full year, from October 2024 to October 2025, allowing the collection of data representing a wide range of seasonal and environmental propagation conditions.

In total, approximately 3.2 million correlation signal samples were recorded, including about 1.5 million during the first half of the campaign (October 2024–March 2025) and around 1.7 million during the second half (April–October 2025). The dataset included correlation results obtained from the VDES R-Mode receiver, containing information on signal delay values and quality indicators. The VDES R-Mode signal [4,17] was transmitted at one-second intervals during the first seven minutes of each hour (during the first month of measurements, the signal was recorded every second; however, due to data recording limitations and the focus of the study being rainy conditions, a scheduled recording scheme was later introduced). This transmission scheme ensured regular and consistent data acquisition throughout the entire observation period. Simultaneously, approximately 110.000 meteorological records were collected by the Milesight WTS506 weather station installed in Jastarnia. The recorded parameters included air temperature, relative humidity, atmospheric pressure, wind direction, wind speed, and rainfall intensity. The data sampling interval was variable and adapted to the prevailing weather conditions: initially, measurements were recorded every 1 min and, then later, every 10 min, and during expected rainfall events, the frequency was increased to 1–3 min to capture rapid atmospheric changes more accurately. Data completeness exceeded 95% of the total observation period, ensuring high temporal consistency between R-Mode signal and environmental measurements.

In parallel with the meteorological data acquisition process, the VDES R-Mode signal was recorded on the receiver side using a software-defined radio platform. The SDR—NI USRP-2954—was used to receive the low-power RF VDES R-Mode signal, convert it to baseband, and forward the complex samples to an industrial computer. The sampling frequency was set to 1 MHz, providing sufficient temporal resolution for precise delay estimation.

The transmitted VDES R-Mode signal followed the VDE-TER frame structure defined for a 100 kHz channel bandwidth with π/4-QPSK modulation [4]. For the applied link configuration, each transmission burst contained 1984 symbols, of which 1877 symbols constituted the net payload used for data transmission and ranging purposes. The ranging signal consisted of a combination of two components: a gold sequence and an alternating sequence. These two sequences were weighted and combined to adapt the ranging performance to different propagation scenarios (short and long distances), providing a compromise between correlation sharpness and robustness to noise and multipath effects.

The recorded in-phase (I) and quadrature (Q) samples were processed by a dedicated signal-processing application developed by the authors. The received signal was subjected to correlation processing using a double-delta correlator, which is an advanced delay estimation technique designed to improve robustness against multipath effects [17]. Compared to a conventional correlator, the double-delta structure enables more accurate determination of signal delay and reduces bias caused by distortions of the correlation function. The estimated signal delay obtained from the correlator output was then used for distance determination and further positioning accuracy analysis.

To automate the measurement process and ensure synchronization between meteorological and signal data, a dedicated data acquisition program was developed in the Node-RED Version 3.0.2 environment [26]. The software was designed using a block-based logical structure, enabling stable operation and efficient management of data flow. It performed a full processing cycle, including reception, decoding, aggregation, storage, and reporting of measurement data. The program operated in several stages:

- Receiving data from LoRa devices—cyclic reception of measurements from the Milesight WTS506 weather station.
- Decoding and processing data—incoming packets were decoded and split into individual parameters: timestamp, battery level, temperature, humidity, wind direction, pressure, wind speed, total rainfall, and rainfall counter. All parameters were then merged into a single object prepared for saving.
- Saving data to a CSV file—decoded data were stored locally as CSV files for further analysis.
- Sending data to the FTP server—every minute, an automated process established a connection with the FTP server and uploaded the latest measurement files.
- Rainfall analysis and aggregation—the system computed total rainfall, deltas between consecutive samples, and hourly rainfall averages.
- Automated daily reporting—every 24 h, a summary report was generated and automatically sent via email using the “trigger 24 h” and “Email Output” nodes.

This setup ensured full automation of data acquisition, verification, and reporting, minimizing the risk of data loss and guaranteeing time alignment between environmental and radio signal observations. Following data acquisition and synchronization, the recorded meteorological parameters were analyzed to determine their temporal variability and representative statistical characteristics. The dataset demonstrated high measurement stability and continuity across all seasons. A summary of the basic statistical characteristics of the recorded meteorological parameters, including minimum, maximum, and mean values, is presented in Table 1.

The compiled dataset covers a complete annual cycle of environmental conditions and provides a reliable foundation for further analysis of the influence of atmospheric parameters—particularly temperature, humidity, and rainfall—on signal propagation and ranging accuracy within the VDES R-Mode system.

### 3.3. Selection of Meteorological Parameters for Further Analysis

Based on the collected measurement data, a preliminary analysis of meteorological variables was carried out to identify the parameters potentially affecting the ranging accuracy of the VDES R-Mode system. The objective was to select environmental factors that exhibit significant temporal variability and may influence radio signal propagation in the VHF band.

From all recorded variables, those directly or indirectly related to tropospheric propagation characteristics were chosen for further consideration. The following parameters were included: air temperature, relative humidity, atmospheric pressure, and rainfall intensity.

A derived parameter—water vapor density—was also calculated in accordance with the ITU-R P.835-7 recommendation [27]. This quantity is commonly used in propagation studies to describe the state of the troposphere and the influence of atmospheric moisture on signal propagation. Unlike relative humidity, which depends on temperature, water vapor density expresses the actual amount of water in the air [g/m^3^], providing a more physically representative description of atmospheric conditions.

The final set of parameters was subsequently used for correlation analysis with ranging accuracy and for the development of a regression model describing the effect of atmospheric conditions on distance measurement performance.

#### 3.3.1. Meteorological Variables Used in the Analysis

The next stage of this study focused on the subset of meteorological parameters identified as relevant in the previous section. These variables were selected based on their statistical behavior in the collected dataset and their potential relationship with variations in distance measurement accuracy. The analysis therefore included air temperature, relative humidity, and rainfall intensity.

In addition, water vapor density—derived from the measured temperature, humidity, and atmospheric pressure—was incorporated into the analysis due to its recognized role in describing atmospheric moisture content in scientific and propagation models.

Parameters associated with air movement, namely wind speed and wind direction, were excluded from further consideration. Preliminary analysis of the collected dataset did not reveal a statistically significant correlation between these parameters and the ranging performance of the VDES R-Mode system. Although wind conditions may influence signal propagation through sea-state-related effects, no consistent or model-relevant relationship between wind parameters and the ranging error was identified in the preliminary data analysis conducted by the authors.

Fog, as one of the meteorological phenomena potentially affecting radio-wave propagation in the maritime VHF environment, could also constitute an interesting parameter for further analysis. However, fog occurrence was not directly recorded by the meteorological station used during the experiment and therefore could not be included as an independent explanatory variable in the present statistical model. It should be noted that parameters related to atmospheric moisture, such as relative humidity and water vapor density, were included in the analysis and may partially reflect conditions associated with fog formation. Nevertheless, a dedicated and explicit assessment of fog influence would require additional monitoring capabilities and is therefore considered a subject for future research.

#### 3.3.2. Determination of Water Vapor Density in the Air According to ITU-R Recommendations

To enable a more accurate relationship between meteorological conditions and ranging accuracy, the water vapor density parameter was introduced into the analysis. This parameter provides a quantitative measure of the water content in the atmosphere and is widely used in propagation and refractivity models. Its determination was carried out in accordance with ITU-R P.835-7, ITU-R P.676-13, and ITU-R P.453-14 recommendations [27,28,29]. The water vapor density was calculated using the relationship given in the following equation:

(5) $$\rho [g/m^{3}]=\frac{e\cdot 216.7}{T},$$

where

e—water vapor pressure [hPa];

T—absolute temperature [K].

The water vapor pressure e was obtained from the relative humidity and saturation vapor pressure values using the formula given in the following equation:

(6) $$e\left[hPa\right]=\frac{RH\cdot e_{s}}{100},$$

where

*RH*—relative humidity [%];

$e_{s}$—saturation vapor pressure [hPa].

The saturation vapor pressure $e_{s}$ depends on temperature and is given by the expression in the following equation:

(7) $$e_{s}[hPa]=EF\cdot a\cdot \exp\left[\frac{\left(b-\frac{t}{d}\right)\cdot t}{t+c}\right],$$

where

EF—enhancement factor;

*a*, *b*, *c*, *d*—empirical constants (*a* = 6.1121, *b* = 18.678, *c* = 257.14, *d* = 234.5).

The enhancement factor EF, which corrects for the non-ideal behavior of moist air, was calculated according to the following equation:

(8) $$EF=1+10^{-4}\left[7.2+P\cdot \left(0.00320+5.9\times 10^{-7}\cdot t^{2}\right)\right],$$

where

P—atmospheric pressure [hPa];

t—temperature [°C].

The above equations form a complete set for determining the water vapor density using temperature, humidity, and pressure data recorded by the monitoring station. This derived parameter was later used in correlation and regression analyses to assess its effect on the accuracy of distance measurements within the VDES R-Mode system.

## 4. Analysis of Ranging Accuracy Errors Obtained During the Measurement Campaign

This section presents an analysis of the distance measurement errors obtained during the long-term ORMOBASS monitoring campaign, with particular focus on the accuracy and stability of VDES R-Mode ranging under real environmental conditions. The assessment focused on the statistical characteristics of distance errors, expressed as root mean square (RMS) values, determined using two reference approaches:

- The hourly precision method, based on short-term statistical variability;
- The reference-based accuracy method, based on errors relative to the reference distance obtained from a one-time system calibration performed at the start of the measurements.

For the reference-based accuracy method, it is important to emphasize that no cumulative error was observed over time. At the beginning of the measurement campaign, precise system calibration was performed using the known geometric distance between the transmitting and receiving stations. As a result, the ranging error relative to the calibrated reference distance remained stable throughout the entire year of observations. Since the true distance was determined with high accuracy, no error accumulation was detected, confirming the long-term stability of the hardware configuration and synchronization scheme.

In the following subsections, the analysis integrates meteorological parameters recorded during the measurement campaign, enabling correlation between propagation conditions and observed RMS values. This approach provides a foundation for identifying environmental factors that have the greatest impact on the stability of distance measurements in the VDES R-Mode system.

### 4.1. Detailed Comparison of RMS Values for Precision and Accuracy Methods

The analysis of ranging error variability was carried out using two approaches corresponding to the hourly precision method and the reference-based accuracy method (i.e., errors calculated relative to the reference distance obtained from a one-time system calibration). In the first approach, the error reference was determined separately for each hourly interval, while in the second approach, a fixed reference distance obtained from the initial system calibration was used for all measurements. In accordance with the measurement scenario, each transmission session lasted approximately seven minutes per hour. During these intervals, distance estimates processed by the correlator of the VDES R-Mode demonstrator were recorded every second. This data structure provided sufficiently high temporal resolution to capture both short-term fluctuations and long-term variations in ranging accuracy. Thanks to the continuous data collection over the entire one-year observation period (from October 2024 to October 2025), it was possible to present the RMS analysis across all twelve months. This comprehensive dataset provides a solid foundation for assessing the long-term stability of the system and for conducting further correlation studies with meteorological conditions.

**Figure 6:**
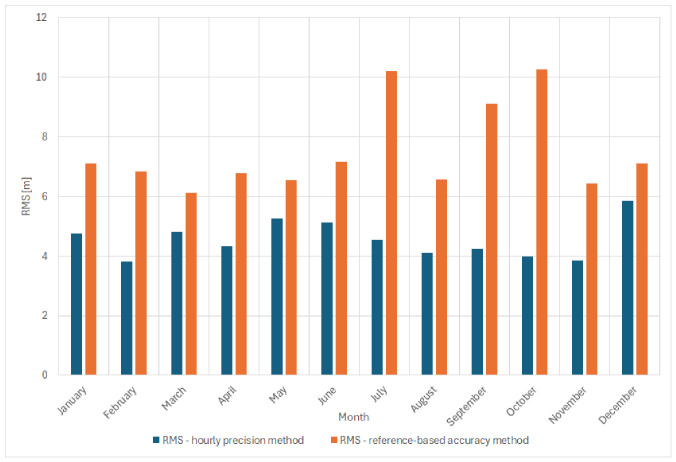
Monthly comparison of RMS distance errors obtained using hourly precision and reference-based accuracy methods.

The comparison of RMS values obtained using both approaches is presented in Figure 6. The results indicate that the RMS calculated with the hourly mean method was consistently lower throughout the entire observation period, with monthly values varying approximately between 3.8 m and 5.9 m, depending on the season. In contrast, the RMS calculated using the reference-based accuracy method resulted in considerably higher monthly values, typically ranging from about 6.1 m to just over 10 m. The smallest differences between the two approaches were observed during the winter months (January–March), whereas during summer and early autumn (July–October), the RMS values increased significantly—particularly for the daily mean method, reaching peak values of around 10.2 m in both July and October.

In addition to the month-by-month comparison, a detailed analysis of selected individual days was also performed. This step served as a preparatory stage for understanding the short-term behavior of the ranging data and for verifying the suitability of the processed results for subsequent regression modeling.

**Figure 7:**
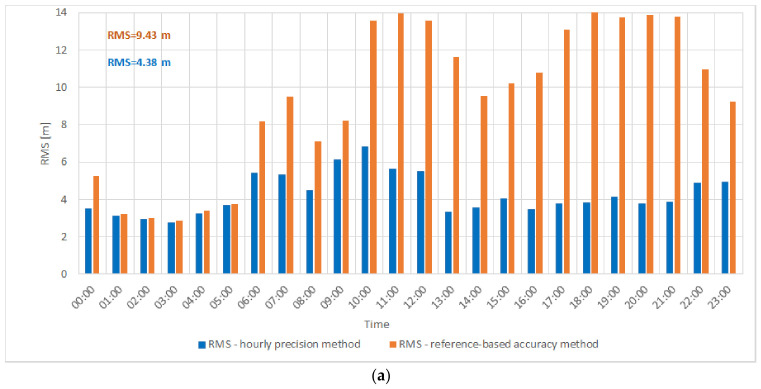

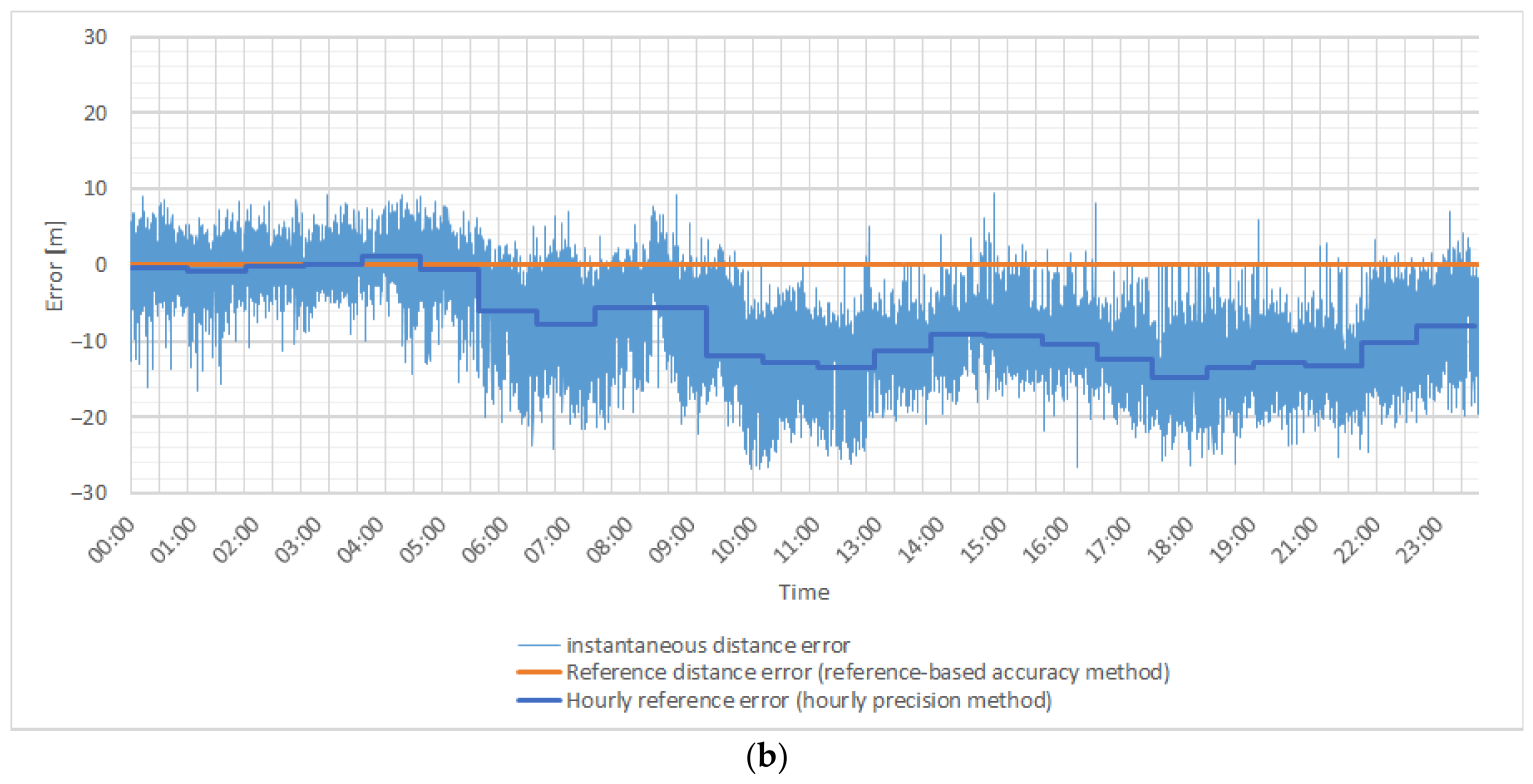
(**a**) Hourly RMS distance errors obtained using the hourly precision method and the reference-based accuracy method (19 October 2024). (**b**) Temporal variation in instantaneous ranging errors and reference error levels over a 24 h period (19 October 2024).

Figure 7a presents the RMS values of distance errors calculated using two different error reference approaches in the VDES R-Mode transmission system for one of the selected days in October. The horizontal axis represents time (hours), while the vertical axis shows the RMS value of the distance error (meters). Orange bars correspond to the reference-based accuracy method, whereas blue bars indicate the hourly precision method.

A comparison of both approaches reveals clear differences. The reference-based accuracy method (orange) results in noticeably higher RMS values throughout the day, particularly during morning and afternoon hours, where the RMS exceeds 10 m. The overall RMS value obtained using this approach was RMS = 9.43 m. In contrast, the hourly precision method (blue) yields lower and more stable RMS values—typically between 2 and 6 m—with an overall value of RMS = 4.38 m, indicating higher precision and effective compensation of short-term variations.

Additionally, the corresponding SNR values were analyzed for the obtained results. It was observed that the short-term RMS values (hourly precision method) were primarily dependent on the measured noise level, since other significant error sources had been minimized or eliminated, as discussed earlier in this paper. In contrast, the RMS values obtained using the reference-based accuracy method, based on errors relative to the calibrated reference distance, may also reflect slow-varying propagation effects, including multipath components caused by atmospheric refraction.

To further illustrate and better understand this relationship, Figure 7b presents the instantaneous distance error relative to the calibrated reference distance for the selected day (19 October 2024), allowing direct observation of short-term error dynamics underlying the RMS differences.

The data presented in Figure 7b exhibit pronounced diurnal fluctuations in distance errors, which can be attributed to variable propagation conditions in the VHF band.

A joint analysis of Figure 7a,b highlights the influence of the adopted error reference method on the resulting RMS values and on the temporal variability of the distance errors. The reference-based accuracy method does not compensate short-term fluctuations, leading to higher RMS values—especially during daytime when instantaneous errors are more dynamic. Conversely, the hourly precision method better reflects local error variability, resulting in lower RMS values and improved short-term ranging precision.

Additionally, Figure 7b reveals an offset of approximately 10 m between the reference-based accuracy and hourly precision reference levels, indicating that the distance error varies over the course of the day. During nighttime, when the propagation environment is more stable and instantaneous errors are smaller, the difference between both methods becomes negligible.

Overall, Figure 7a,b confirm that the ranging performance of the VDES R-Mode system depends not only on instantaneous signal stability and propagation conditions but also on the adopted error reference method. The hourly precision method proves more effective in representing time-varying effects and provides a more realistic representation of short-term distance measurement precision.

### 4.2. Integration of Meteorological Data with Signal Samples Processed by the VDES R-Mode Demonstrator Correlator

As part of the subsequent analytical phase, meteorological parameters were mapped to the signal samples processed by the correlator of the VDES R-Mode demonstrator. The primary objective of this step was to create a unified dataset combining ranging accuracy metrics with environmental variables, enabling a detailed assessment of how atmospheric conditions affect distance measurement accuracy.

Both data sources—ranging results and meteorological observations—were recorded independently and at different temporal resolutions. The VDES R-Mode correlator produced distance estimates every second during each seven-minute transmission session, while the weather-station data were collected at variable sampling intervals, ranging from one to ten minutes depending on local conditions.

To ensure temporal consistency, all datasets were synchronized based on their timestamps. The synchronization procedure included the averaging of high-frequency records and the removal of incomplete, inconsistent, or outlier samples.

The integration process was implemented using a dedicated data-processing script, which automatically matched each correlation sample with the corresponding set of meteorological parameters. This approach enabled the creation of a single structured database containing, for each timestamp, the following fields: distance error, air temperature, relative humidity, atmospheric pressure, rainfall intensity, and water vapor density.

The resulting dataset ensured precise temporal alignment between signal measurements and atmospheric parameters, which is essential for accurate correlation and regression analyses.

**Figure 8:**
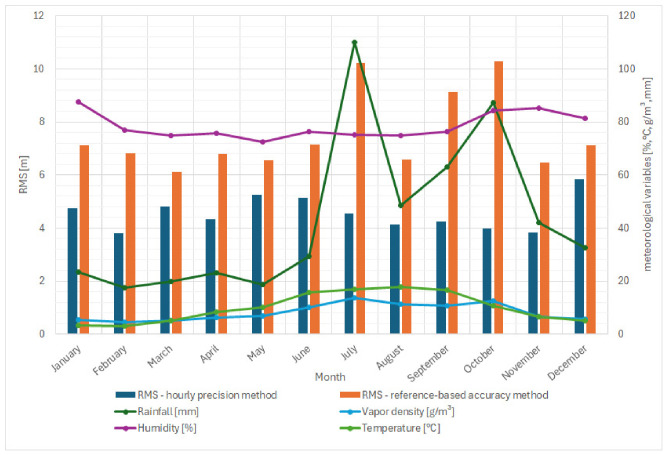
Monthly averaged RMS values and corresponding meteorological parameters derived from long-term measurements.

A summary of the averaged monthly values of key parameters is presented in Figure 8, including both RMS indicators (calculated using the hourly precision method and the reference-based accuracy method), meteorological measurements, and derived quantities such as water vapor density (ρ). The averaged values shown in this figure were obtained by aggregating the results discussed in Section 4.1 (Detailed Comparison of RMS Values for Precision and Accuracy Methods) and mapping the corresponding integrated meteorological parameters onto a monthly timescale.

The underlying dataset comprises more than 110.000 meteorological records collected over the one-year observation period, ensuring high temporal consistency with the VDES R-Mode signal samples. A preliminary inspection of the results suggests a potential relationship between rainfall intensity and water vapor density, with higher RMS values typically observed during the summer months. This relationship is examined in detail in subsequent sections, where the influence of meteorological conditions on ranging accuracy is analyzed using statistical methods and regression modeling.

### 4.3. Detailed Comparison of RMS Values Depending on Rainfall Conditions

To assess the influence of weather conditions on ranging accuracy, the collected data were analyzed on a daily scale. This allowed the comparison of instantaneous distance errors and RMS values with the corresponding meteorological parameters such as rainfall intensity, humidity, and temperature. The goal of this analysis was to identify potential correlations between environmental variability and variations in the ranging accuracy of the VDES R-Mode system.

**Figure 9:**
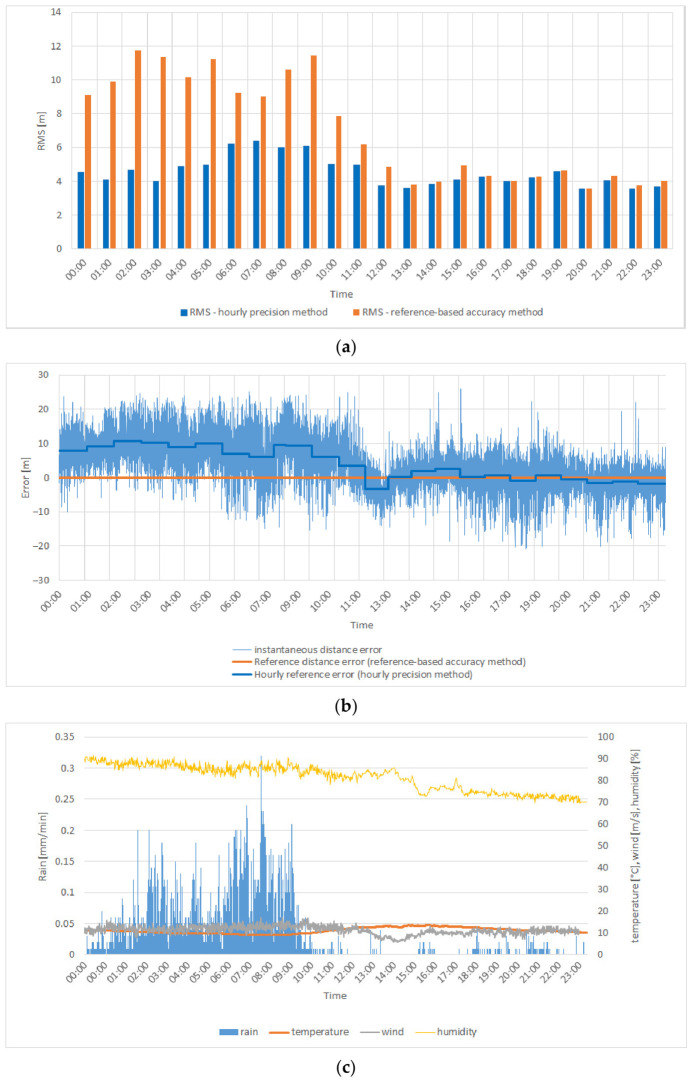
(**a**) Hourly RMS distance errors obtained using hourly precision and reference-based accuracy methods (3 November 2024). (**b**) Temporal variation in instantaneous ranging errors and reference error levels over a 24 h period (3 November 2024). (**c**) Meteorological conditions recorded during the selected day (3 November 2024), including rainfall intensity, relative humidity, air temperature, and wind speed.

Figure 9a–c present the results obtained for 3 November 2024, illustrating the relationship between ranging error variability and the corresponding meteorological conditions during the analyzed rainy day.

During the night and morning hours (00:00–11:00), a distinct increase in RMS values is observed, particularly for the reference-based accuracy method. In this period, intensive rainfall occurred, as indicated in Figure 9c, while humidity remained close to 100%. Although the RMS values obtained using the hourly precision method remained relatively stable—averaging around 4–5 m—the RMS values obtained using the reference-based accuracy method increased significantly, reaching more than 10 m. This divergence suggests that rainfall increased the temporal variability of the signal, resulting in an overall increase in the RMS error relative to the calibrated reference distance. The elevated RMS during precipitation events may indicate that rainfall adversely affects the stability of the propagation path and the consistency of distance measurements, introducing additional noise and multipath effects. After 11:00, when rainfall ceased and humidity began to decrease, the RMS values gradually declined, confirming that the presence of precipitation correlates with increased ranging errors. This observation indicates that rainfall acts as a destabilizing factor for the VDES R-Mode ranging system, increasing the overall distance measurement error despite relatively stable short-term fluctuations. The presented case represents one of many analyzed days showing similar relationships. Across multiple datasets, a noticeable influence of rainfall on changes in RMS values obtained using the reference-based accuracy method was observed. Further statistical analysis of the complete dataset will allow verification of whether other meteorological parameters—such as humidity, temperature, or atmospheric pressure—also had a measurable impact on ranging accuracy.

### 4.4. Statistical Analysis and Correlation Between RMS and Meteorological Parameters

Rainfall was identified as the dominant environmental factor already during the preliminary data processing stage, where its significant influence on distance measurement errors was clearly observed. For this reason, it was analyzed in detail in Section 4.3. The purpose of this subsection is to provide a comparative statistical assessment of all considered meteorological parameters, including rainfall, within a unified framework, supporting the subsequent regression-based modeling.

Based on the full dataset collected over the one-year monitoring campaign, an extended statistical analysis was carried out to examine how the accuracy of distance measurements (expressed through RMS values) changes under varying meteorological conditions. The purpose of this analysis was to identify whether specific atmospheric parameters systematically influence the ranging performance of the VDES R-Mode system. Understanding these relationships is essential for developing predictive models capable of compensating for environmental effects and improving ranging accuracy, especially in real-time maritime navigation scenarios.

The analysis was performed by grouping all integrated samples according to selected meteorological parameter intervals—rainfall intensity, air temperature, relative humidity, and water vapor density. For each bin, the mean RMS value and statistical indicators were computed. This approach enabled the identification of potential monotonic relationships and nonlinear behaviors that may not be apparent from single-day case studies. Table 2 presents a summarized and illustrative mapping of RMS values against the corresponding average meteorological conditions for each parameter bin. The table is intended to provide a general overview of observed tendencies.

The results reveal several important trends. First, rainfall intensity exhibited a clear influence on RMS behavior. The RMS values for non-rain conditions remained significantly lower compared with intervals characterized by even light rainfall, and they increased further for moderate and heavy precipitation. This trend is consistent across the entire dataset, suggesting that rainfall—even at low intensities—acts as a disturbance mechanism that modifies the propagation conditions in the VHF band, resulting in increased measurement error.

A similar relationship was observed for water vapor density, whose growth correlates with a noticeable rise in RMS error. For water vapor density above approximately 5–11 g/m^3^, the RMS values show a clear increasing tendency, which suggests that the total content of atmospheric moisture, not only relative humidity, plays a measurable role in radio-wave propagation variability.

The influence of temperature and relative humidity appears weaker and less monotonic, although certain tendencies are still present. RMS values tend to grow at lower temperatures and at very high relative humidity levels, which may indicate additional effects such as increased refractivity gradients in colder, moist layers of the troposphere.

Overall, the statistical results presented in the tables demonstrate that rainfall intensity and water vapor density show the strongest and most consistent relationship with RMS error. These findings provide the first quantitative basis for further modeling: the detected dependencies suggest that a regression-based predictive model—particularly one using a logarithmic form—may capture these relationships effectively. The development and validation of such a model is presented in the next section.

## 5. Modeling Distance Measurement Error Using Logarithmic Regression

The statistical analysis presented in the previous section demonstrated that selected meteorological parameters—particularly rainfall intensity and water vapor density—exert a measurable influence on the ranging accuracy of the VDES R-Mode system. These findings highlight the need for a predictive model capable of representing the relationship between atmospheric conditions and the observed RMS ranging error. Such a model would enable the estimation of accuracy degradation under varying environmental scenarios and could serve as the basis for real-time correction mechanisms implemented directly within future R-Mode receivers.

An approach based on statistical modeling of meteorological influences has already been successfully applied in other radio-based systems, where environmental parameters were treated as explanatory variables describing performance degradation. For example, regression-based models have been used to quantify the impact of temperature and humidity on received signal strength in outdoor wireless sensor networks, demonstrating that weather-dependent effects can be effectively captured using data-driven methods derived from long-term measurements [30]. Similarly, in terrestrial radio systems operating at higher frequencies, such as millimeter-wave fixed links, rainfall intensity has been explicitly modeled as a dominant factor affecting signal attenuation, with empirical and semi-empirical models employed to predict weather-induced performance degradation [31]. These studies confirm that statistical modeling of meteorological conditions constitutes a valid and established methodology for characterizing environmental effects on radio systems.

The model developed in this study is intended as a practical tool, with its performance to be verified through a dedicated measurement campaign within the ORMOBASS project framework. The validation phase will utilize a mobile receiver installed on a ferry operating in the Baltic Sea, thereby enabling evaluation under dynamically varying geometry and propagation distances.

Based on these established approaches, the objective of this section is to develop and evaluate a mathematical model that captures the dependence of the RMS ranging error on key meteorological variables in the VDES R-Mode system. During the preliminary stage of the study, linear, logarithmic, and polynomial regression formulations were implemented and evaluated within the developed software environment. The polynomial formulation was rejected at an early stage due to its unsuitable modeling behavior and limited physical interpretability for the analyzed atmospheric relationships. The linear regression model produced slightly lower $R^{2}$ (coefficient of determination) values compared to the logarithmic formulation. Given the nonlinear nature of the observed relationships, the logarithmic regression model ultimately provided the most stable and physically meaningful representation of the dependence between meteorological conditions and ranging error. In addition, the adopted formulation offers low computational complexity and straightforward interpretation, which are desirable features for potential practical implementation in maritime VHF positioning systems. The following sections present the methodology, parameter selection, model calibration procedure, and quantitative assessment of the model’s performance, leading to a formulation that effectively represents the environmental contribution to ranging inaccuracies.

### 5.1. Multivariate Logarithmic Regression Model

The results obtained in the preceding analysis indicate that the ranging error of the VDES R-Mode system (expressed through RMS values) exhibits a clear and systematic dependence on selected meteorological parameters, particularly rainfall intensity and water vapor density. These relationships are inherently nonlinear: for low values of a parameter, the error changes slowly, whereas above a certain threshold, it increases much more rapidly. Such behavior is characteristic of logarithmic models, where the logarithmic transformation allows both the gradual growth and the sharp increases to be represented within a single functional form.

The starting point is the general linear regression model, in which the expected ranging error is described as a linear combination of selected explanatory variables and a stochastic component [32,33,34]. In its basic form, the model can be written as

(9) $$E\approx a_{0}+a_{1}x_{1}+a_{2}x_{2}+\cdots +a_{K}x_{K}+\varepsilon ,$$

where

E—expected ranging error (e.g., RMS);

$x_{1},x_{2},\ldots ,x_{K}$—selected meteorological parameters (temperature, humidity, rainfall, and water vapor density);

$a_{0},a_{1},\ldots ,a_{K}$—regression coefficients;

ε—random error term representing unmodeled effects.

In logarithmic regression, the model remains linear with respect to the coefficients, but the explanatory variables are replaced by their logarithms. For a single variable, such as rainfall intensity *R*, the model becomes

(10) $$E\approx a_{0}+a_{1}\ln\left(R\right),$$

while for multiple variables, the model can be expressed as given in the following equation:

(11) $$E\approx a_{0}+a_{1}\ln\left(x_{1}\right)+a_{2}\ln\left(x_{2}\right)+\cdots +a_{K}\ln\left(x_{K}\right).$$

Although nonlinear in terms of the input variables, the model is linear in the coefficients $a_{K}$. This property allows the use of standard least-squares estimation. The nonlinearity is handled entirely by transforming the variables before constructing the regression matrix. In the implemented software, this transformation is applied selectively depending on user settings in the interface.

Assuming a dataset of N observations, each containing the measured ranging error and a corresponding set of meteorological parameters, the data can be represented in vector form, as given in the following equation,

(12) $$y=\left[\begin{array}{c} E_{1}\\ E_{2}\\ .\\ .\\ .\\ E_{N} \end{array}\right],$$

and the design matrix is defined as given in the following equation:

(13) $$X=\;\left[\begin{array}{ccccc} 1 & z_{11} & z_{12} & \cdots & z_{1K}\\ 1 & z_{21} & z_{22} & \cdots & z_{2K}\\ \vdots & \vdots & \vdots & & \vdots \\ 1 & z_{N1} & z_{N2} & \cdots & z_{NK} \end{array}\right],$$

where

$z_{iK}=x_{iK}$ for linear regression;

$z_{iK}=ln(x_{iK})$ for logarithmic regression.

The vector of regression coefficients is given in the following equation:

(14) $$a=\left[\begin{array}{c} a_{0}\\ a_{1}\\ .\\ .\\ .\\ a_{K} \end{array}\right],$$

The model can then be written compactly, as given in the following equation:

(15) y≈Xa+ε.

The least-squares solution minimizes the sum of squared residuals, as given in the following equation:

(16) $$S\left(a\right)={\left|\left|y-Xa\right|\right|}^{2}.$$

Setting the derivative of S with respect to a to zero leads to normal equations:

(17) $$X^{T}Xa=X^{T}y.$$

which, in matrix form, leads to the following equation:

(18) $$\left[\begin{array}{ccccc} \sum 1 & \sum z_{i1} & \sum z_{i2} & \cdots & \sum z_{iK}\\ \sum z_{i1} & \sum z_{i1}z_{i1} & \sum z_{i1}z_{i2} & \cdots & \sum z_{i1}z_{iK}\\ \sum z_{i2} & \sum z_{i1}z_{i2} & \sum z_{i2}z_{i2} & \cdots & \sum z_{i2}z_{iK}\\ \vdots & \vdots & \vdots & & \vdots \\ \sum z_{iK} & \sum z_{i1}z_{iK} & \sum z_{i2}z_{iK} & \cdots & \sum z_{iK}z_{iK} \end{array}\right]\cdot \left[\begin{array}{c} a_{0}\\ a_{1}\\ a_{2}\\ \vdots \\ a_{K} \end{array}\right]=\left[\begin{array}{c} \sum E_{i}\\ \sum {E_{i}z}_{i1}\\ \sum {E_{i}z}_{i2}\\ \vdots \\ \sum {E_{i}z}_{iK} \end{array}\right]$$

Provided that $X^{T}X$ is invertible, the solution is given in the following Equation (19):

(19) $$a=\;{\left(X^{T}X\right)}^{-1}X^{T}y.$$

Once the coefficient vector is estimated, the modeled ranging error for each sample is computed as given in the following equation:

(20) $$\hat{E_{i}}\approx a_{0}+a_{1}z_{i1}+a_{2}z_{i2}+\cdots +a_{K}z_{iK}.$$

Model performance is evaluated using the coefficient of determination $R^{2}$, as defined in the following equation:

(21) $$R^{2}=1-\frac{{SS}_{err}}{{SS}_{tot}},$$

where

$${SS}_{err}=\sum_{i=1}^{N}{\left(E_{i}-\hat{E_{i}}\right)}^{2},$$

$${SS}_{tot}=\sum_{i=1}^{N}{\left(E_{i}-\bar{E}\right)}^{2},$$

with $\bar{E}$ denoting the sample mean of the measured errors. Values of $R^{2}$ close to 1 indicate strong explanatory power of the model.

A second key metric is the residual root-mean-square error, as defined in the following equation:

(22) $$RMS=\sqrt{\frac{1}{N}\sum_{i=1}^{N}{\left(E_{i}-\hat{E_{i}}\right)}^{2}}.$$

In the context of VDES R-Mode ranging, RMS is directly interpretable in meters, making it an intuitive measure for comparing different model configurations—single-variable, two-variable, three-variable, linear, or logarithmic.

In summary, the multivariate logarithmic regression model developed in this work relies on assembling a design matrix from selected meteorological parameters (in linear or logarithmic form), solving the normal equations using least squares, and evaluating the resulting model using $R^{2}$ and RMS metrics. The next section describes the practical implementation of this methodology in the dedicated software tool, which allows dynamic selection of input variables and real-time assessment of model performance.

To enable practical use of the logarithmic regression model developed in the previous subsection, a dedicated software tool was implemented in the C++ programming language using the C++ Builder environment. The goal of the application is to process meteorological data, construct regression models, and analyze the quality of model fitting in relation to the distance measurement errors recorded during the VDES R-Mode monitoring campaign.

The software operates on CSV files containing both meteorological parameters and ranging error measurements collected throughout the one-year observation period. These inputs include temperature, relative humidity, rainfall intensity, and derived quantities such as water vapor density. The program integrates numerical methods, automated data management, and an interactive graphical interface, which supports efficient exploration of regression configurations. The application performs automated preprocessing of input data, including numerical conversion, consistency checks, and filtering based on predefined meteorological constraints. As part of this stage, water vapor density is calculated for each observation using a formulation consistent with ITU-R recommendations, providing a unified and quality-controlled dataset for further analysis. Based on user-defined selections, the software constructs regression models of varying dimensionality, supporting both linear and logarithmic formulations. The regression parameters are estimated using the classical normal-equation approach, and model performance is evaluated using standard statistical metrics, including RMS error and the coefficient of determination (*R*^2^). An interactive graphical user interface enables flexible configuration of predictor variables, real-time inspection of intermediate results, and visual comparison between measured and modeled ranging errors. In addition to standard regression analysis, the tool supports interval-based and nested statistical evaluations, allowing detailed investigation of the influence of individual meteorological parameters and their combined effects on ranging accuracy.

### 5.2. Variable Selection and Final Logarithmic Regression Model Formulation

The selection of variables for the logarithmic regression model was guided by the statistical analyses presented in the preceding sections of this article, which examined the relationship between meteorological conditions and RMS ranging error over the full one-year observation period. These analyses consistently indicated that rainfall intensity exhibits the strongest and most systematic influence on distance measurement accuracy in the VDES R-Mode system, making it the primary explanatory variable for further modeling.

Having established rainfall as the dominant factor, additional analyses were conducted to identify a complementary parameter capable of improving the descriptive capability of the regression model. Several atmospheric variables were evaluated, including air temperature, relative humidity, dynamic humidity thresholds, and water vapor density. While temperature and humidity showed only weak or inconsistent relationships with the ranging error, water vapor density demonstrated a noticeably stronger and more stable association. This behavior reflects its physical relevance, as vapor density inherently combines the effects of air temperature and moisture content and is directly linked to atmospheric refractivity.

Based on these findings, rainfall intensity and water vapor density were selected as the predictors for the final multivariate logarithmic regression model. The final comparison was performed for the water vapor density range of 5.33–11.02 g/m^3^ and rainfall intensities between 0.01 and 0.4 mm/min, as these intervals provided the best combined RMS and *R*^2^ performance. The results are summarized in Table 3.

Table 3 presents the coefficients of determination obtained for single-, two-, and three-parameter logarithmic regression models. Among single-parameter configurations, rainfall intensity yields the highest coefficient of determination (*R*^2^ = 0.48), followed by water vapor density (*R*^2^ = 0.28), while air temperature alone exhibits only a marginal contribution (*R*^2^ = 0.11). These results confirm the dominant role of precipitation in shaping the observed ranging error variability.

Extending the model to two parameters leads to a substantial improvement when rainfall is combined with water vapor density, yielding *R*^2^ = 0.57. This value represents the highest explanatory capability among all tested configurations. In contrast, the inclusion of temperature in multi-parameter models results in only a negligible increase in explanatory power. The three-parameter model combining temperature, water vapor density, and rainfall achieves *R*^2^ = 0.58, indicating that the additional contribution of temperature is limited and largely redundant.

Consequently, the two-parameter logarithmic regression model combining rainfall intensity and water vapor density was selected as the final formulation, as it offers the best balance between model complexity and explanatory strength. The selected model is expressed as given in the following equation:

(23) $$Error\left[m\right]=11.42+1.37\cdot \ln\left(V\right)+2.84\cdot \ln\left(R\right),$$

where

*V*—$vapor\;density\;[g/m^{3}]$;

*R*—rainfall [mm/min],

with performance metrics

$$\begin{array}{c} RMS\left[m\right]=3.51,\\ R^{2}=0.57, \end{array}$$

where the model is valid for the following ranges of vapor density and rainfall intensity:

$$\begin{array}{c} 5.33\;<\;V\;<\;11.02\;,\\ 0.01\;<\;R\;<\;0.4\;, \end{array}$$

and the propagation distance between the transmitter and receiver = 19.9 km,

$$H_{tx}=28\;m\;a.s.l.\;and\;\;H_{rx}=17\;m\;a.s.l.$$

**Figure 10:**
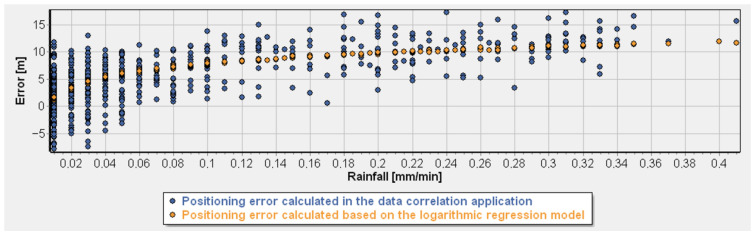
Comparison of measured and modeled ranging errors for the final logarithmic regression model.

These values confirm the robustness of the selected formulation and demonstrate its ability to capture the dominant atmospheric mechanisms affecting distance determination accuracy. The behavior of the final model is illustrated in Figure 10 (the plot was generated using logarithmic regression modeling software developed by the authors), which shows good agreement between measured and modeled ranging errors, particularly under conditions of elevated atmospheric moisture and active precipitation.

Due to the temporal nature of the collected dataset, characterized by strong autocorrelation of both meteorological and ranging error time series, an additional statistical verification was performed using heteroskedasticity and autocorrelation consistent (HAC) standard errors, estimated according to the Newey–West method [35]. In this approach, the covariance matrix of the estimated regression coefficients, consistent with the notation adopted earlier in this article, is expressed as

(24) $$Var\left(\hat{a}\right)={\left(X^{T}X\right)}^{-1}S_{u}{\left(X^{T}X\right)}^{-1},$$

where

$S_{u}$—the covariance matrix incorporating weighted autocovariances of residuals.

The HAC-consistent standard error of the *i*-th coefficient was then obtained as

(25) $${SE}_{HAC}\left({\hat{a}}_{i}\right)=\sqrt{{Var\left(\hat{a}\right)}_{ii}},$$

where

${SE}_{HAC}\left({\hat{a}}_{i}\right)$—the HAC-consistent standard error of the *i*-th regression coefficient;

${Var\left(\hat{a}\right)}_{ii}$—the *i*-th diagonal element of the HAC covariance matrix.

The statistical significance of individual regression coefficients was then evaluated using t-statistics [33] defined as

(26) $$t_{i}=\frac{{\hat{a}}_{i}}{{SE}_{HAC}\left({\hat{a}}_{i}\right)},$$

where

$t_{i}$—t-statistic for the *i*-th coefficient.

The results of this analysis are summarized in Table 4.

The results indicate that rainfall remains a statistically significant predictor even after accounting for autocorrelation effects, while the contribution of water vapor density appears less robust, which is consistent with the earlier correlation analysis presented in this study.

In addition, to further assess the stability of the model under reduced temporal dependence, a sparse sampling approach was applied by selecting every 10th observation from the dataset. The resulting model performance was characterized by

$$\begin{array}{c} RMS\left[m\right]=3.59,\\ R^{2}=0.501, \end{array}$$

indicating that the model retains its predictive capability even under reduced data density. The re-estimated model obtained after sparse sampling validation confirms that the identified relationships are not solely driven by short-term temporal correlations but reflect underlying physical dependencies between atmospheric conditions and ranging error. In particular, the rainfall-related component remained highly consistent after sparse re-estimation, while the contribution of water vapor density remained physically coherent, although less pronounced compared to the full-sample model. The obtained results confirm that it is feasible to construct a compact and physically justified mathematical description of atmospheric impacts on distance measurement accuracy in radio-based maritime positioning systems. In particular, precipitation rate and atmospheric water content emerge as the key environmental drivers of ranging error variability in the VDES R-Mode system.

### 5.3. Assessment of Model Fit Quality and Model Validation

**Figure 11:**
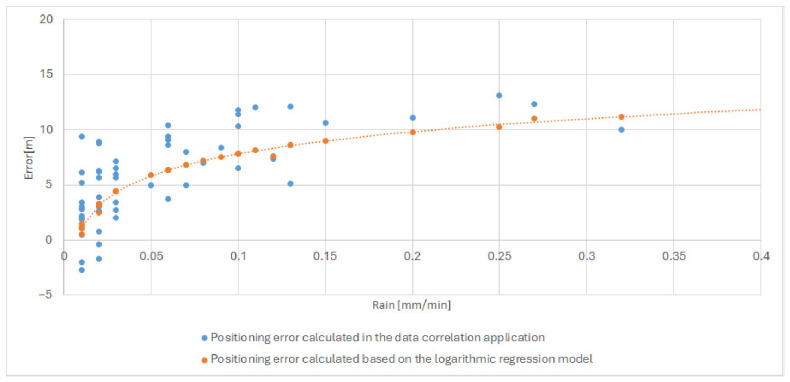
Use of the final logarithmic regression model for correcting ranging errors under rainy conditions (28 July 2025, dataset used in model training).

After selecting the two-parameter logarithmic regression model based on rainfall intensity and water vapor density, the next step was to evaluate its practical usefulness by applying it to real measurement data from a rainy day. The goal of this validation was to verify whether the developed model can effectively correct distance determination errors and thus improve ranging accuracy under adverse atmospheric conditions. Figure 11 presents the selected regression model superimposed on a dataset corresponding to one of the rainy measurement periods (selected date in July 2025–28 July 2025), which was included in the training dataset. The distance error is defined as the difference between the measured distance and a fixed reference distance determined at the beginning of the measurement campaign; therefore, both positive and negative values may occur, corresponding to overestimation and underestimation of the true distance. Additionally, the fitted logarithmic regression model is indicated in the figure using a dashed line.

**Figure 12:**
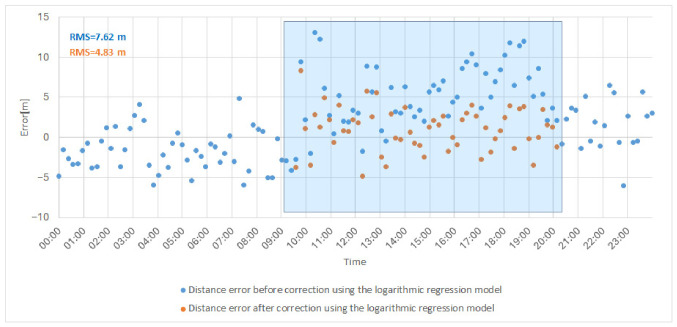
Application of the logarithmic regression-based correction in the time domain during a rainy measurement period (28 July 2025, dataset used in model training).

The agreement between the modeled and measured values confirms that the selected model captures the dominant trend in how rainfall affects ranging accuracy. To further analyze this effect in the time domain, Figure 12 presents the ranging error with the model-based correction applied. The blue-shaded region in the figure indicates the time interval during which rainfall was recorded by the meteorological station. The predicted error values obtained from the logarithmic regression model are used to compensate for the measured distance error. As shown, the corrected error distribution becomes significantly narrower, reducing the amplitude of deviations associated with rainfall. The resulting reduction in RMS from 7.62 m (uncorrected) to 4.83 m demonstrates a substantial improvement in ranging accuracy. This nearly 40% reduction provides clear evidence that the proposed model effectively captures and mitigates the rainfall-induced component of the ranging error. Consequently, the remaining error is primarily associated with noise and other minor environmental influences.

**Figure 13:**
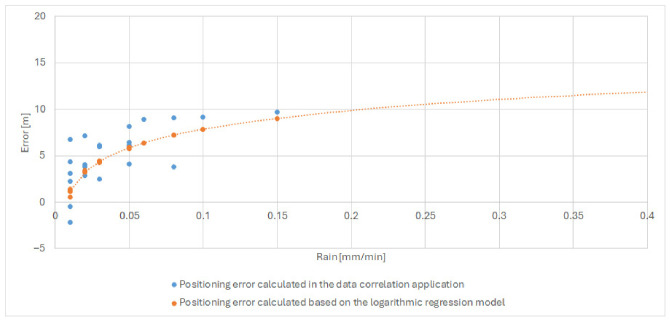
Use of the final logarithmic regression model for correcting ranging errors under rainy conditions (14 November 2025, independent validation dataset).

In the next step, the model was evaluated using an independent dataset not included in the training process. The results are presented in Figure 13, which shows the application of the final logarithmic regression model to a rainy measurement period on 14 November 2025. This date was selected as it represents a rainy day meeting the conditions under which the model is applicable.

**Figure 14:**
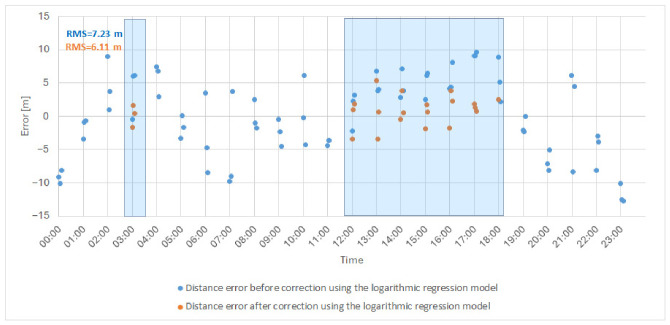
Application of the logarithmic regression-based correction in the time domain during a rainy measurement period (14 November 2025, independent validation dataset).

For the uncorrected data, an RMS value of 7.23 m was obtained, while the application of the model-based correction reduced this value to 6.11 m. The time-domain behavior of the corrected error is presented in Figure 14. A clear decrease in error is observed during the rainfall period, indicating that the model effectively compensates for the influence of both precipitation and water vapor density on the ranging error under independent conditions.

These results confirm the practical efficiency of the selected logarithmic regression model. When applied to real measurement data, the model demonstrably reduces error variability during rainy periods, functioning as a dynamic correction mechanism. This suggests that, in real-time applications, such a model could be integrated into the VDES R-Mode processing pipeline to enhance the stability and reliability of distance estimates under challenging meteorological conditions.

## 6. Conclusions

This study demonstrates that the development of a mathematical model capable of representing and compensating atmospheric effects on distance determination accuracy in the VDES R-Mode system is both feasible and practically justified. Based on a comprehensive one-year dataset of synchronized ranging measurements and meteorological observations, the authors have shown that selected atmospheric parameters—most notably rainfall intensity and water vapor density—exert measurable influence on VDES R-Mode ranging performance.

The conducted analyses confirm that, although the proposed logarithmic regression model is valid within defined meteorological ranges (e.g., rainy conditions and specific intervals of water vapor density), such limitations are inherent and fully acceptable in radio-propagation modeling. A similar modeling philosophy is adopted in well-established ITU-R recommendations, where dedicated empirical or semi-empirical models are formulated for specific propagation mechanisms and environmental conditions. This particularly includes the well-known ITU-R P.838-3, ITU-R P.676, and ITU-R P.530 recommendations. In this context, the logarithmic regression model proposed in this article should be interpreted as a complementary correction mechanism tailored to specific atmospheric scenarios—most notably precipitation-driven propagation disturbances—rather than as a universal solution applicable under all conditions. This approach is consistent with the established ITU-R methodology and reflects standard practice in the development of operationally useful propagation models.

A key outcome of this work is the verification that meaningful correction of atmospheric-induced ranging errors can be achieved without continuous real-time monitoring of all VDES R-Mode reference stations. While monitoring stations remain an effective tool for performance supervision, their deployment and maintenance significantly increase system cost and complexity. The results presented in this article indicate that, for certain operating conditions, mathematical models derived from long-term observations can partially replace or support monitoring-based correction schemes, offering a more cost-effective and scalable alternative for future VDES R-Mode deployments.

This study deliberately focused on long-term stationary measurements conducted under stable line-of-sight conditions over a maritime path—thereby minimizing both small- and large-scale fading effects, as well as other error sources discussed at the beginning of this article. This choice ensured that the observed error variations were dominated by atmospheric effects rather than geometric changes or multipath variability, thereby providing a clean and reliable basis for model development. The authors acknowledge that this represents a favorable scenario; however, it constitutes a necessary first step in verifying whether atmospheric correction modeling is possible at all. The effectiveness of the selected two-parameter logarithmic regression model—based on rainfall intensity and vapor density—was confirmed through validation on independent rainy-day datasets. The demonstrated reduction in RMS ranging error obtained during both training-dataset evaluation and independent validation confirms the practical potential of the proposed model as a dynamic correction mechanism that can be integrated directly into VDES R-Mode receivers without consuming additional radio resources.

The findings presented in this article also support ongoing standardization activities. The authors continue to contribute to international R-Mode standardization efforts within IALA and IMO working groups, ensuring that the results of the ORMOBASS project directly inform the development of future maritime navigation frameworks. Future work will focus on extending the validation of the proposed modeling approach to dynamic scenarios.

In summary, this study confirms that atmospheric-effect modeling for VDES R-Mode ranging accuracy is not only theoretically sound but also practically applicable. Similar to correction models long employed in satellite navigation systems and propagation models used in terrestrial radio systems, the proposed approach demonstrates that environment-driven performance variations in ground-based maritime radionavigation can be effectively addressed using dedicated, scenario-specific models. Building on the authors’ prior experience in VDES system development and VHF radio-channel modeling in complex propagation environments, including offshore wind farms, this work establishes atmospheric modeling as a viable complementary tool that can reduce system complexity, enhance robustness, and support the cost-effective deployment of resilient maritime positioning systems.

## Figures and Tables

**Table 1:** Basic statistical characteristics of meteorological parameters recorded during the measurement campaign.

	Mean	Min	Max
Temperature [°C]	11.0	−4.9	26.8
Relative Humidity [%]	80.5	31.0	99.5
Wind Speed [m/s]	5.6	0	22.8
Atmospheric Pressure [hPa]	1018.8	987.8	1040.6
Rainfall [mm/min]	0.05	0	0.41

**Table 2:** Statistical summary of RMS distance errors for different meteorological parameter intervals.

RMS by Rainfall Intensity Bins				
Rain [mm/min] Bin	RMS Error [m]	Mean Temp [°C]	Mean Humidity [%]	Mean *ρ* [g/m^3^]
0 (no rain)	8.18	10.13	61.52	7.16
0–0.01	7.22	11.58	76.75	8.1
0.01–0.05	7.87	10.76	73.7	7.31
0.05–0.10	8.92	10.91	71.9	7.22
0.10–0.20	9.24	10.81	72.38	7.23
0.20–0.40	10.93	11.68	77.13	8.15
>0.40	15.63	15.2	68	8.84
RMS by temperature bins				
Temp [°C] bin	RMS error [m]	Mean temp [°C]	Mean humidity [%]	Mean $\rho \;[g/m^{3}]$
<0	11.30	−1.27	84.77	3.78
0–5	8.01	3.21	87.16	5.28
5–10	7.05	8.41	83.63	7.13
10–15	5.71	11.55	81.17	8.42
15–20	7.96	16.51	73.25	10.29
>20	7.22	20.92	60.61	11.03
RMS by humidity bins				
Hum [%] bin	RMS error [m]	Mean temp [°C]	Mean humidity [%]	Mean $\rho \;[g/m^{3}]$
0–60	6.09	11.67	54.75	5.92
60–75	6.28	11.13	69.57	7.13
75–85	6.37	10.32	79.93	7.79
>85	8.63	9.28	91.27	8.31
RMS by water vapor density bins				
$\rho \;[g/m^{3}]\;$bin	RMS error [m]	Mean temp [°C]	Mean humidity [%]	Mean $\rho \;[g/m^{3}]$
0–5	6.96	3.89	71.69	4.44
5–8	7.41	10.26	78.45	6.93
8–11	8.81	12.71	82.45	9.11
>11	9.56	18.04	82.52	12.68

**Table 3:** Coefficients of determination for logarithmic regression models evaluated for selected meteorological parameter ranges.

Number of Parameters	Parameters	*R* ^2^
1	temperature	0.11
1	vapor density	0.28
1	rainfall	0.48
2	temperature, rainfall	0.50
2	vapor density, rainfall	0.57
3	temperature, vapor density, rainfall	0.58

**Table 4:** Regression coefficients and corresponding HAC-based t-statistics.

Parameter	Coeff (${\hat{a}}_{i}$)	*t_i_*
const (${\hat{a}}_{0}$)	11.42	3.67
rainfall (${\hat{a}}_{1}$)	2.84	15.83
vapor density (${\hat{a}}_{2}$)	1.37	1.96

## Data Availability

All collected measurement data and simulation results analyzed during the current study are available from the corresponding author on reasonable request.
